# ECOLOGICAL MOMENTARY ASSESSMENT OF SUICIDE RISK: FEASIBILITY AMONG OLDER ADULTS WITH MEMORY CONCERNS

**DOI:** 10.1093/geroni/igae098.1836

**Published:** 2024-12-31

**Authors:** Taylor Loskot, Emily Bower

**Affiliations:** Pacific University, Hillsboro, Oregon, United States; Pacific University, Hillsboro, Oregon, United States

## Abstract

Given heightened suicide risk among older adults,, effective and timely risk assessment strategies are imperative to inform suicide prevention. Ecological momentary assessment (EMA) may help detect fluctuations in socioemotional suicide risk factors, including loneliness, depression, and suicide ideation (SI). The aim of the present study was to examine feasibility of an EMA battery for assessing momentary SI and socioemotional risk factors in older adults with subjective cognitive decline (SCD). Participants (n=27; M age=69; 82% female) were recruited from a larger study and were classified into “higher risk” (n=13) and “lower risk” (n=14) groups based on history of suicidal behavior or recent SI. Using an EMA app on their smartphone, participants were notified to complete four surveys daily for 14 days. EMA items included questions about SI, mood, loneliness, and cognitive concerns. After the survey period, participants completed validated “paper-and-pencil” assessments for comparison and provided feedback about app usability. On average, participants completed two-thirds of assigned surveys (M = 37, SD = 10) and completion rates did not vary significantly by suicide risk, (t(25) = 0.73, p >.05) or SCD severity (r = -.11, p >.05). Participants reported an easy and non-disruptive experience and provided helpful feedback regarding the structure of notifications, survey wording, and app interface. Additional demographic and psychosocial predictors of EMA response rate will be explored in regression models. Overall positive feedback suggests that EMA may be a feasible risk assessment method for use with older adults facing cognitive concerns.

